# Evaluation of the clinical outcomes of the *Test and Treat* strategy to implement *Treat All* in Nigeria: Results from the Nigeria Multi-Center ART Study

**DOI:** 10.1371/journal.pone.0218555

**Published:** 2019-07-10

**Authors:** Kristen A. Stafford, Solomon F. Odafe, Julia Lo, Ramat Ibrahim, Akipu Ehoche, Mercy Niyang, Gambo G. Aliyu, Bola Gobir, Dennis Onotu, Ademola Oladipo, Ibrahim Dalhatu, Andrew T. Boyd, Otse Ogorry, Lawal Ismail, Manhattan Charurat, Mahesh Swaminathan

**Affiliations:** 1 Department of Epidemiology and Public Health, University of Maryland, Baltimore, Maryland, United States of America; 2 Institute of Human Virology, University of Maryland School of Medicine, Baltimore, Maryland, United States of America; 3 Centers for Disease Control and Prevention, CGH/DGHT, Abuja, Nigeria; 4 Maryland Global Initiatives Corporation, Abuja, Nigeria; 5 Department of Medicine, University of Maryland School of Medicine, Baltimore, Maryland, United States of America; 6 Centers for Disease Control and Prevention, CGH/DGHT, Atlanta, Georgia, United States of America; 7 PEPFAR Coordination Office, Abuja, Nigeria; 8 Walter Reed Army Institute of Research, Military HIV Research Program, Abuja, Nigeria; University of Ghana College of Health Sciences, GHANA

## Abstract

In December 2016, the Nigerian Federal Ministry of Health updated its HIV guidelines to a *Treat All* approach, expanding antiretroviral therapy (ART) eligibility to all individuals with HIV infection, regardless of CD4+ cell count, and recommending ART be initiated within two weeks of HIV diagnosis (i.e., the *Test and Treat* strategy). The *Test and Treat* policy was first piloted in 32 local government areas (LGAs). The primary objective of this study was to evaluate the clinical outcomes of adult patients initiated on ART within two weeks of HIV diagnosis during this pilot. We conducted a retrospective cohort analysis of patients who initiated ART within two weeks of new HIV diagnosis between October 2015 and September 2016 in eight randomly selected LGAs participating in the *Test and Treat* pilot study. 2,652 adults were newly diagnosed and initiated on ART within two weeks of HIV diagnosis. Of these patients, 8% had documentation of a 12-month viral load measurement, and 13% had documentation of a six-month viral load measurement. Among *Test and Treat* patients with a documented viral load, 79% were suppressed (≤400 copies/ml) at six months and 78% were suppressed at 12 months. By 12 months post-ART initiation, 34% of the patients who initiated ART under the *Test and Treat* strategy were lost to follow-up. The median CD4 cell count among patients initiating ART within two weeks of HIV diagnosis was 323 cells/mm^3^ (interquartile range, 161–518). While randomized controlled trials have demonstrated that *Test and Treat* strategies can improve patient retention and increase viral suppression compared to standard of care, these findings indicate that the effectiveness of *Test and Treat* in some settings may be far lower than the efficacy demonstrated in randomized controlled trials. Significant attention to the way *Test and Treat* strategies are implemented, monitored, and improved particularly related to early retention, can help expand access to ART for all patients.

## Introduction

HIV continues to have a major impact on public health globally with an estimated 36.9 million people living with HIV (PLWH) including 1.8 million new infections in 2017 [1]. Beginning in 2013, the Coordinating Board of the Joint United Nations Programme on HIV/AIDS (UNAIDS) called upon UNAIDS to set targets with the goal of ending the AIDS epidemic globally. In response, the 90-90-90 strategy was announced in 2014, laying out an ambitious worldwide target of 90% of all PLWH knowing their status, 90% of these PLWH receiving sustained antiretroviral therapy (ART), and 90% of all PLWH on ART achieving durable viral suppression by 2020 [2]. Achieving these targets is particularly important for epidemic control in sub-Saharan Africa, which remains disproportionately affected by the epidemic. At the end of 2017, 59% of PLWH in sub-Saharan Africa were receiving ART, an increase from 24% in 2010 [1]. The expansion of access to ART also resulted in a 36% reduction in AIDS-related deaths in the region over the same period [1]. These gains, however, have not been equally distributed over all countries in the region.

Nigeria has the second largest HIV epidemic globally [3]. While new HIV infections in Nigeria have decreased 21% since 2010, only 30% of the estimated 3.2 million PLWH in Nigeria have access to ART, and AIDS-related deaths have decreased only 6% since 2010 [3]. Over the past several years, studies have demonstrated clear benefits for treating PLWH early in disease progression [4]. Studies have shown that immediate initiation of ART after a positive HIV test result could contribute to epidemic control [5–8], prompting the launch of the World Health Organization *Treat All* guidelines in 2016 [9]. HIV treatment sites supported by the United States President’s Emergency Plan for AIDS Relief (PEPFAR) in Nigeria piloted the *Test and Treat* strategy of initiating ART within two weeks of HIV diagnosis as part of the *Treat All* policy in 32 local government areas (LGAs) beginning in October 2015. To expand the *Treat All* approach from piloted LGAs to sites nationwide and to decrease the time to initiating ART after a positive HIV test result, the Federal Ministry of Health of Nigeria made two significant changes to their national HIV prevention and treatment guidelines in December 2016. First, CD4+ cell count thresholds for ART initiation were removed, thus expanding ART eligibility to all HIV-positive individuals, and second, ART should be initiated as soon as possible following HIV diagnosis (preferably within two weeks) [10].

To evaluate the outcomes of the *Test and Treat* pilot, which was implemented just before the changes in national guidelines, we conducted a retrospective cohort study of patients who initiated ART within two weeks of HIV diagnosis during the pilot. The primary objectives of this study were to evaluate viral suppression and ART retention among adult patients who initiated ART within two 2 weeks of HIV diagnosis in a sample of the pilot LGAs. A secondary objective of this study was to compare these outcomes with those of patients previously diagnosed and enrolled in care in the same LGAs. Additional secondary analyses included demographic and clinical predictors of loss to follow-up (LTFU) or non-adherence to ART, time to LTFU, and odds of non-adherence to ART across the first year of treatment.

## Methods

### Study design

We conducted a retrospective cohort analysis of patients who initiated ART between October 2015 and September 2016 in eight LGAs randomly selected from the 32 which participated in the *Test and Treat* pilot across five states in Nigeria.

### Study population

The study populations consisted of two groups of adult patients. The first group included newly identified HIV-positive patients who initiated ART within two weeks of diagnosis irrespective of clinical or immunological status (the *Test and Treat* group). The second group included were HIV-positive patients enrolled in care before the *Test and Treat* pilot who then initiated ART irrespective of immunologic or clinical status during the study period. To be eligible for the study, patients had to be aged at least 16 years at the time of ART initiation and had to have initiated ART at one of the eligible sites.

### Sample size

To achieve a final sample representative of the population that initiated ART during the *Test and Treat* pilot, we estimated a study sample size of 3,432 patients; when corrected for 20% projected missing medical records, the target *Test and Treat* sample size was 4,290 patients. This sample size was calculated assuming 75% retention, 5% precision of estimates, and a design effect of 3.0 to account for the clustering of the sampling method at the LGA level.

### Sampling method

We used a three-stage probability proportionate to size sampling method. First, 25% (8) of the 32 LGAs were selected with a probability of selection proportionate to the number of total patients receiving ART within each LGA, according to the 2015 PEPFAR Annual Progress Report (APR). Second, we included all facilities in each of the eight selected LGAs that had at least 50 patients receiving ART according to the 2015 PEPFAR Annual Progress Report that had implemented *Test and Treat* for adult patients, and that had offered ART services before October 2015. For the final stage of the sampling, we multiplied the total sample size by the facility selection probability proportional to the total number of patients meeting the inclusion criteria at the facility. A sampling frame was then created of all patients meeting the inclusion criteria using patients’ unique identification numbers as reported electronically by the facilities using the Retention and Audit Determination Tool and divided by the sample size for the site to provide the interval for systematic sampling of medical records to be used after the first randomly selected identification number until the number required from the site was complete. If a medical record was missing, the next medical record on the list of randomly ordered unique identification numbers was selected.

### Data collection and quality assurance

We manually abstracted the following data from the electronic and paper medical records at the sampled facilities: outcomes of ART, demographic variables, clinical variables including functional status, HIV diagnosis information, ART initiation and subsequent visits, regimen information, baseline and subsequent laboratory measurements including viral load and CD4 cell count, co-infections and opportunistic infections, ART refill data, and support services received. All data abstractors were trained to understand the survey objectives, their roles in the evaluation, best practices for good data quality, the importance of confidentiality, and the survey instruments. Each data abstraction team had a dedicated, onsite supervisor during abstraction to perform quality checks.

### Human subjects research protection

The evaluation study protocol and abstraction surveys were approved by the National Health Research Ethics Committee Nigeria and the Institutional Review Board of the University of Maryland, Baltimore. Because the study was retrospective, the requirements for written informed consent were waived. The study was reviewed according to the United States Centers for Disease Control and Prevention (CDC) human research protection procedures and was determined to be, and approved as, research, but CDC was not engaged.

### Statistical analysis

All variables were initially investigated in univariate analysis to determine frequency, distribution, and the amount of missing data for each variable. Patients were grouped by ART initiation strategy: newly diagnosed and initiated on ART within two weeks (Test and Treat), and previously diagnosed and enrolled in care.

Viral load measurements for the 6-month outcome were eligible for inclusion if they were measured between 4 and 8 months after ART initiation. Viral load measurements for the 12-month outcome were eligible for inclusion if they were measured between 10 and 14 months after ART initiation. In our analysis, we defined viral suppression as a viral load test result of less than 400 HIV ribonucleic acid copies/mL, per Nigerian integrated national guidelines for HIV prevention, treatment, and care [10]. We also estimated viral suppression using the World Health Organization (WHO)-recommended level of less than 1000 copies/mL for comparison.

LTFU was defined as no documented attendance at the health care facility within 90 days from the date of the missed scheduled visit to see a health care provider or to pick up medication. Kaplan-Meier methods were used to estimate the time to LTFU among Test and Treat patients by relevant clinical and demographic variables. The constancy of care engagement after ART initiation was calculated as the proportion of quarters in which a patient had at least one visit in the first year of ART [11]. The first 14 days of follow-up were excluded to avoid overestimation of first quarter retention.

Because of the frequency of missing viral load results, we used medication refill data to determine adherence to ART. Non-adherence to refill was defined as an interval between medication refills when the ratio between the number of days of pills dispensed by days between refills was less than 95%. Patients had to have at least two documented refill dates and the number of pills dispensed documented at the previous refill for inclusion in the non-adherence to refill analysis. Refills in the first 14 days of ART were excluded. Days between refill visits were calculated for all patients with a refill date and corresponding number of days’ worth of pills dispensed. The adherence rate was calculated as the (number of days’ worth of pills dispensed divided by the days between refills) multiplied by 100 [12]. Time from documented ART initiation date was used to calculate the refill visit interval for the first 12 months of treatment. Generalized estimating equations to account for the correlation between repeated measurements were used to model the odds of non-adherence at each interval. The observed and model-based results were then plotted with corresponding 95% confidence intervals (CIs). A sensitivity analysis was conducted excluding all observations in the first 30 days to determine the potential influence of low levels of early non-adherence on the model-based slope of non-adherence over time.

To estimate the rate of LTFU among *Test and Treat* patients, we estimated cause-specific hazards for LTFU using Cox proportional hazards with a random effect for facility and with a frailty term in the models. Patients who died or who were transferred were excluded from this analysis due to the low numbers available for a competing risks analysis. To identify independent predictors of LTFU, we used generalized linear models allowing for a binary outcome distribution using stepwise selection. Predicted probabilities were then calculated for each patient on the basis of the independent predictors and then were plotted on a receiver operator characteristics curve to calculate a c-statistic to estimate the ability of the combination of predictors to discriminate a patient who was LTFU from one who was retained. Statistical analysis was conducted using STATA 15 (StataCorp LLC, College Station TX) and SAS 9.4 (SAS Institute, Cary NC).

## Results

### Demographic and clinical characteristics of study population

The final study sample included 3,256 PLWH whose medical records were abstracted from 31 clinics across eight LGAs. Of these, 2,652 (81%) were *Test and Treat* patients. The remaining 604 (19%) were in the previously diagnosed and enrolled in care group. Of the patients included in the final study sample, 91% were missing documentation of a 12-month viral load measurement, and 86% were missing documentation of a six-month viral load measurement.

Across the study sample, the median age at ART enrolment was 33 years (interquartile range (IQR), 27–40 years). The median CD4+ cell count at ART initiation was 323 cells/mm^3^ (IQR, 161–518). The sample was 68% female and 50% married. Eighty-five percent of HIV-positive individuals in the study population did not have their partners’ HIV status documented in the medical records. Of those with partner status documentation, 49% of patients reported having an HIV-positive partner (Table 1).

**Table 1:** Baseline characteristics of “Test and Treat” evaluation study patients initiated on antiretroviral therapy between October 1, 2015 and September 30, 2016 in randomly selected Local Government Areas in Nigeria by antiretroviral therapy initiation strategy (N = 3,256).

Characteristic		Total	Test & Treat	Previously Diagnosed and Enrolled in Care	P-value
		(N = 3,256)	(n = 2,652) n (%)	(n = 604) n (%)	
**Sex**					0.11
	Female	2,202	1777 (67.0)	425 (70.4)	
	Male	1,054	875 (33.0)	179 (29.6)	
**Age**					0.05
	16–24	489	420 (15.8)	69 (11.4)	
	25–34	1,322	1073 (40.5)	249 (41.2)	
	35–44	949	752 (28.4)	197 (32.6)	
	45–54	363	298 (11.2)	65 (10.8)	
	>55	133	109 (4.1)	24 (4.0)	
**Marital Status**					0.28
	Single	819	673 (29.3)	146 (27.7)	
	Married	1,643	1322 (57.6)	321 (60.9)	
	Divorced	41	32 (1.4)	9 (1.7)	
	Widowed	223	192 (8.4)	31 (5.9)	
	Other	97	77 (3.4)	20 (3.8)	
	Missing	433	356	77	
**Education**					<0.0001
	None	381	320 (17.5)	61 (16.4)	
	Primary School	468	400 (21.8)	68 (18.3)	
	Secondary School	845	713 (38.9)	132 (35.5)	
	Post-Secondary	454	367 (20.0)	87 (23.4)	
	University	38	17 (0.9)	21 (5.6)	
	Other	18	15 (0.8)	3 (0.8)	
	Missing	1,052	820	232	
**Employment Status**					<0.0001
	Employed	1,614	1253 (58.8)	361 (70.2)	
	Unemployed	1,032	879 (41.2)	153 (29.8)	
	Missing	610	520	90	
**Partner HIV Status**					0.05
	Negative	257	219 (53.4)	38 (42.2)	
	Positive	243	191 (46.6)	52 (57.8)	
	Missing	2,756	2,242	514	
**Pregnant**					0.013
	Yes	149	131 (5.3)	18 (3.1)	
	No	1,826	1451 (59.1)	375 (65.6)	
	NA	1,054	875 (35.6)	179 (31.3)	
	Missing	227	195	32	
**BMI**					<0.0001
	Underweight	396	360 (17.0)	36 (7.2)	
	Normal range	1,545	1,254 (59.3)	291 (58.3)	
	Overweight	674	502 (23.7)	172 (34.5)	
	Missing	641	536	105	
**CD4 Level**					<0.0001
	<200 cells/mm^3^	609	541 (33.1)	68 (19.9)	
	201–350 cells/mm^3^	462	402 (24.6)	60 (17.6)	
	351–500 cells/mm^3^	378	309 (18.9)	69 (20.2)	
	> 500 cells/mm^3^	526	382 (23.4)	144 (42.2)	
	Missing	1,281	1,018	263	
**WHO Stage**					<0.0001
	I	2,068	1661 (64.5)	407 (70.3)	
	II	516	402 (15.6)	114 (19.7)	
	III	522	471 (18.3)	51 (8.8)	
	IV	49	42 (1.6)	7 (1.2)	
	Missing	101	76	25	
**Functional Status**					0.004 ‡
	Ambulatory	89	83 (3.3)	6 (1.1)	
	Bedridden	11	11 (0.4)	0 (0.0)	
	Working	2,958	2431 (96.3)	527 (98.9)	
	Missing	198	127	71	
**ARV Regimen**					0.09
	XTC-TDF-EFV	3,090	2519 (96.5)	571 (96.1)	
	3TC-AZT-NVP	85	64 (2.5)	21 (3.5)	
	Other	29	27 (1.0)	2 (0.3)	
	Missing	52	42	10	
**STI**					0.11
	No	2,191	1809 (97.0)	382 (98.5)	
	Yes	62	56 (3.0)	6 (1.5)	
	Missing	1,003	787	216	
**STI List**					0.41‡
	Chlamydia	3	3 (6.0)	0 (0.0)	
	Gonorrhea	1	1 (2.0)	0 (0.0)	
	Herpes	2	1 (2.0)	1 (16.7)	
	Syphilis	6	6 (12.0)	0 (0.0)	
	Others	44	39 (78.0)	5 (83.3)	
	Missing	8	8	0	
**Hepatitis C**					0.12‡
	Negative	240	176 (92.6)	64 (98.5)	
	Positive	15	14 (7.4)	1 (1.5)	
	Missing	3,001	2,462	539	
**TB Status**					0.002
	No Sign of TB	2,508	2,004 (82.3)	504 (89.0)	
	Prior History of TB Tx	443	391 (16.1)	52 (9.2)	
	On TB Treatment	51	41 (1.7)	10 (1.8)	
	Missing	254	216	38	
**Chronic Illness**					0.20
	No	2,197	1815 (96.6)	382 (95.3)	
	Yes	83	64 (3.4)	19 (4.7)	
	Missing	976	773	203	
**Cotrimoxazole**					0.58
	No	295	237 (9.2)	58 (9.9)	
	Yes	2,876	2348 (90.8)	528 (90.1)	
	Missing	85	67	18	
**Pre-ART Counselling**					0.80
	No	98	79 (3.4)	19 (3.6)	
	Yes	2,789	2276 (96.6)	513 (96.4)	
	Missing	369	297	72	
**ART Initiation Counselling**					0.01
	No	120	88 (3.7)	32 (6.1)	
	Yes	2,782	2290 (96.3)	492 (93.9)	
	Missing	354	274	80	

‡ Fisher’s test

Compared with patients who were previously diagnosed and enrolled in care, *Test and Treat* patients were more likely to be aged 16–24 years (p = 0.05), to have only primary or secondary school education (p<0.0001), to be unemployed (p<0.0001), to report having an HIV-negative partner (p = 0.05), to be underweight (p<0.0001), to have a baseline CD4+ cell count <200 cells/mm^3^ (p<0.0001), to be WHO stage III (p<0.0001), to have an ambulatory functional status (p = 0.004), to have a prior history of TB treatment (p<0.0001), and to have received ART initiation counseling (p = 0.01); Among women in the study population, those with new HIV diagnoses (*Test and Treat*) were more likely to be pregnant (p = 0.013). The median number of days to ART initiation after enrolment in the facility among *Test and Treat* patients was 0 days (IQR, 0–3 days).

### Viral suppression at 6 and 12 months

Of the 348 *Test and Treat* patients that had a 6-month viral load result, 79% had viral suppression (≤400 copies/mL); 79% of the 107 previously enrolled patients with 6-month viral load results also had viral suppression. Of the 204 *Test and Treat* patients with a 12-month viral load result, 78% were suppressed (≤400 copies/mL), compared to 84% of the 75 previously enrolled patients with a documented 12-month viral load result. There was no evidence of a difference in viral suppression outcomes at 6 months and 12 months when comparing *Test and Treat* patients to those previously enrolled in care (Table 2). When using the WHO-recommended viral threshold of 1,000 copies/mL, there was no evidence of a difference in suppression between *Test and Treat* patients and previously enrolled patients at six months, and lower suppression among Test and Treat patients compared to previously diagnosed patients at 12 months.

**Table 2:** On treatment viral suppression at six and 12 months after the initiation of antiretroviral therapy among patients initiated on treatment between October 1, 2015 and September 30, 2016 in Nigeria by ART initiation strategy.

**Viral suppression at 6-months: per-protocol analysis (≤ 400 copies/ml)**					
	**Total**		**Viral Suppression**	**No Viral Suppression**	**P-value**
	**(n = 455)**		**(n = 358)**	**(n = 97)**	** **
Test & Treat	348		274 (78.7)	74 (21.3)	0.96
Previously Diagnosed and Enrolled in Care	107		84 (78.5)	23 (21.5)	
**Viral suppression at 6-months: sensitivity analysis (≤ 1,000 copies/ml)**					
	**Total**		**Viral Suppression**	**No Viral Suppression**	**P-value**
	**(n = 455)**		**(n = 378)**	**(n = 77)**	** **
Test & Treat	348		288 (82.8)	60 (17.2)	0.74
Previously Diagnosed and Enrolled in Care	107		90 (84.1)	17 (15.9)	
**Viral suppression at 12-months: per- protocol analysis (≤ 400 copies/ml)**					
** **	**Total**	**Viral Suppression**		**No Viral Suppression**	**P-value**
** **	**(n = 279)**	**(n = 222)**		**(n = 57)**	** **
Test & Treat	204	159 (77.9)		45 (22.1)	0.27
Previously Diagnosed and Enrolled in Care	75	63 (84.0)		12 (16.0)	
**Viral suppression at 12-months: sensitivity analysis (≤ 1,000 copies/ml)**					
	**Total**	**Viral Suppression**		**No Viral Suppression**	**P-value**
	**(n = 279)**	**(n = 234)**		**(n = 45)**	** **
Test & Treat	204	166 (81.4)		38 (18.6)	0.06
Previously Diagnosed and Enrolled in Care	75	68 (90.7)		7 (9.3)	

Viral suppression varied by LGA from 67% to 91%. Higher baseline CD4 levels were significantly associated with viral suppression at 6 months (89% and 84% for patients with a baseline CD4 cell count of 351–500 cells/mm^3^ and >500 cells/mm^3^, respectively, compared to 70% and 75% for patients with a baseline CD4 cell count of <200 cells/mm^3^ and 201–350 cells/mm^3^, respectively. No variables were significantly associated with viral suppression at 12 months.

### Vital status at end of follow-up among Test and Treat patients

A total of 21,338 patient months were observed among *Test and Treat* patients. Of the 2,652 *Test and Treat* patients, 42 (2%) died, 128 (5%) were transferred out from the health facilities, and 903 (34%) were LTFU, compared to five (1%), 21 (3%), and 116 (19%) of the previously diagnosed patients, respectively (Table 3). Of the *Test and Treat* patients LTFU, 51% were LTFU within the first 30 days following ART initiation compared to 30% among the previously diagnosed patients (p<0.0001).

**Table 3:** Patient outcomes comparing patients initiated on antiretroviral therapy within two weeks of an initial HIV-positive test to patients previously diagnosed and enrolled in care prior to antiretroviral therapy initiation between October 1, 2015 and September 30, 2016 in Nigeria (n = 3,256).

		Total	Died	Alive	Transferred Out	Stopped ART	Lost to Follow Up	Lost to Follow Up in the first 30 days^a^	P-value
		(n = 3,256)	(n = 47)	(n = 2,039)	(n = 149)	(n = 2)	(n = 1,019)	(n = 497)	
**ART Initiation Strategy**									<0.0001
	Test& Treat, n (%)	2,652	42 (1.6)	1579 (59.5)	128 (4.8)	0 (0.0)	903 (34.1)	462 (51.2)	
	Previously Diagnosed and Enrolled in Care, n (%)	604	5 (0.8)	460 (76.2)	21 (3.5)	2 (0.3)	116 (19.2)	35 (30.2)	

^a^Lost to follow up in the first 30 days is a subset of all lost to follow up and proportions are calculated using total lost to follow up within comparison group as the denominator

### Retention consistency among Test and Treat patients

Of the 2,652 *Test and Treat* patients, 1,193 (45%) had at least one visit in each quarter of the first year of treatment: 411 (15%), 249 (9%), 340 (13%), and 459 (17%) had visits in three, two, one, and no quarters, respectively. Lower visit constancy was associated with an increased likelihood of LTFU (p<0.0001).

### Adherence to refill among Test and Treat patients

**Fig 1:**
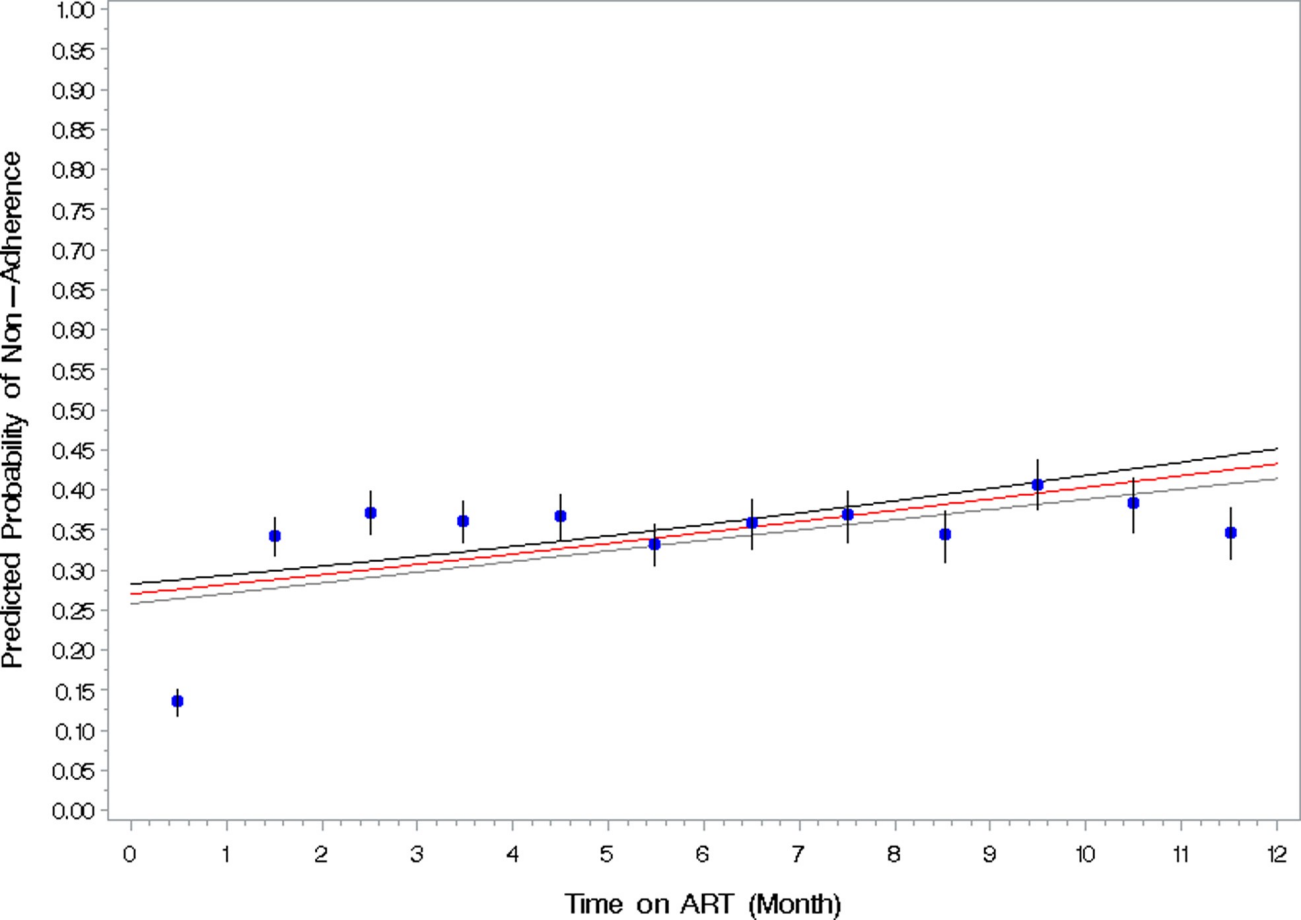
Model based probability of non-adherence to refill in the first 12 months of antiretroviral therapy among newly diagnosed patients initiated on treatment within two weeks of HIV diagnosis (n = 2,206).

Adherence to refill was calculable for 11,520 observations among *Test and Treat* patients. Of the 2,652 *Test and Treat* patients, 2,206 had at least one prescription with a corresponding number of pills dispensed and two documented refill dates. Of the total refills observed, 67% had pills to cover at least 95% of their treatment days, 10% had 85%–94% of their treatment days covered, 5% had 75%–84% of their treatment days covered, and 18% had less than 75% of their treatment days covered. The number of patients with optimal adherence to refills (≥95%, which indicates that patients have enough pills for each day between refills) decreased in the second month of treatment. In other words, non-adherence to refill was significantly higher in the second month compared with the first month. Model-based estimates of non-adherence demonstrate an increasing trend over time. Observed non-adherence to refill fluctuated from month 3 to 12, but there was no clear trend. The proportion of patients with optimal adherence remained below 70% following the first month of treatment (Fig 1). These proportions do not include patients who died, who were LTFU, or who never received a refill after their initial prescription. The results of the sensitivity analysis, excluding observations in the first 30 days of ART, indicated that non-adherence after 1 month through 12 months is fairly constant at approximately 35% and did not increase over time. Therefore, in the full model including the first month of ART, non-adherence is likely underestimated for the first six months of treatment.

### Factors associated with LTFU among Test and Treat patients

Of the *Test and Treat* patients, 903 were LTFU by 12 months after their ART initiation date. Among these patients, the crude rate of LTFU in the *Test and Treat* population was 3.97/100 person-months on ART (95% CI, 3.61–4.33). Higher LTFU was observed for younger patients (46% for those aged 16–24 years of age vs. 33% for those aged >55 years; p<0.0001), those who were divorced or single compared with those who were married (p<0.0001), those with a secondary education or lower compared to those with post-secondary education or above (p<0.0001), those unemployed compared to employed patients (p = 0.01), patients who were underweight compared to those who were normal or overweight at the time of ART initiation (p<0.0001), those with higher WHO clinical stage compared to those with lower WHO stage (p<0.0001), and those with an ambulatory or bed-ridden functional status compared with those with a working status (p = 0.01), those with prior history of tuberculosis compared to those without tuberculosis or those currently receiving tuberculosis treatment (p = 0.02), those without documented receipt of cotrimoxazole (p = 0.003), and those without documented pre-ART counseling (p = 0.009; Table 4).

**Table 4:** Factors associated with loss to follow up among patients initiated on antiretroviral therapy within two weeks of diagnosis of HIV infection in Nigeria (n = 2,482).

Characteristic		Total	Not Lost to Follow Up	Lost to Follow Up	P-value
		(N = 2,482)	(n = 1,579) n (%)	(n = 903) n (%)	
**Sex**					0.87
	Female	1,668	1063 (63.7)	605 (36.3)	
	Male	814	516 (63.4)	298 (36.6)	
**Age**					<0.0001
	16–24	394	212 (53.8)	182 (46.2)	
	25–34	1,006	642 (63.8)	364 (36.2)	
	35–44	709	468 (66.0)	241 (34.0)	
	45–54	276	192 (69.6)	84 (30.4)	
	>55	97	65 (67.0)	32 (33.0)	
**Marital Status**					<0.0001
	Single	621	345 (55.6)	276 (44.4)	
	Married	1,246	845 (67.8)	401 (32.2)	
	Divorced	30	21 (70.0)	9 (30.0)	
	Widowed	175	125 (71.4)	50 (28.6)	
	Other	69	49 (71.0)	20 (29.0)	
	Missing	341	194	147	
**Educational Status**					<0.0001
	None	293	188 (64.2)	105 (35.8)	
	Primary School	364	217 (59.6)	147 (40.4)	
	Secondary School	660	395 (59.9)	265 (40.2)	
	Post-Secondary	344	258 (75.0)	86 (25.0)	
	University	17	15 (88.2)	2 (11.8)	
	Other	13	10 (76.9)	3 (23.1)	
	Missing	791	496	295	
**Employment Status**					0.01
	Employed	1,191	799 (67.1)	392 (32.9)	
	Unemployed	794	485 (61.1)	309 (38.9)	
	Missing	497	295	202	
**Partner HIV Status**					0.32
	Negative	210	167 (79.5)	43 (20.5)	
	Positive	181	151 (83.4)	30 (16.6)	
	Missing	2,091	1,261	830	
**Pregnancy Status**					0.60
	No	1,351	867 (64.2)	484 (35.8)	
	N/A	814	516 (63.4)	298 (36.6)	
	Yes	128	87 (68.0)	41 (32.0)	
	Missing	189	109	80	
**BMI**					<0.0001
	Underweight	325	191 (58.8)	134 (41.2)	
	Normal range	1,170	788 (67.4)	382 (32.7)	
	Overweight	481	351 (73.0)	130 (27.0)	
	Missing	506	249	257	
**CD4**					0.25
	<200 cells/mm^3^	505	375 (74.3)	130 (25.7)	
	201–350 cells/mm^3^	381	274 (71.9)	107 (28.1)	
	351- 500cells/mm^3^	302	211 (69.9)	91 (30.1)	
	> 500cells/mm^3^	366	250 (68.3)	116 (31.7)	
	Missing	928	469	459	
**WHO Stage**					<0.0001
	I	1,593	1099 (69.0)	494 (31.0)	
	II	362	231 (63.8)	131 (36.2)	
	III	419	201 (48.0)	218 (52.0)	
	IV	35	21 (60.0)	14 (40.0)	
	Missing	73	27	46	
**Functional Status**					0.011‡
	Ambulatory	76	40 (52.6)	36 (47.4)	
	Bedridden	9	3 (33.3)	6 (66.7)	
	Working	2,277	1482 (65.1)	795 (34.9)	
	Missing	120	54	66	
**First ARV Regimen**					0.98
	XTC-TDF-EFV	2,359	1504 (63.8)	855 (36.2)	
	3TC-AZT-NVP	60	39 (65.00)	21 (35.0)	
	Other	27	17 (63.0)	10 (37.0)	
	Missing	36	19	17	
**TB Status**					0.02
	No Sign of TB	1,900	1263 (66.5)	637 (33.5)	
	Prior history of TB	344	202 (58.7)	142 (41.3)	
	On TB Treatment	36	25 (69.4)	11 (30.6)	
	Missing	202	89	113	
**Chronic Illness**					0.32
	No	1,700	1133 (66.6)	567 (33.4)	
	Yes	59	43 (72.9)	16 (27.1)	
	Missing	723	403	320	
**Cotrimoxazole**					0.003
	No	216	118 (54.6)	98 (45.4)	
	Yes	2,203	1430 (64.9)	773 (35.1)	
	Missing	63	31	32	
**Pre-ART Counselling**					0.009
	No	77	39 (50.6)	38 (49.4)	
	Yes	2,121	1380 (65.1)	741 (34.9)	
	Missing	284	160	124	
**ART Initiation Counselling**					0.21
	No	84	49 (58.3)	35 (41.7)	
	Yes	2,133	1385 (64.9)	748 (35.1)	
	Missing	265	145	120	

‡ Fisher's test

Rates of LTFU decreased as patient age increased and among patients who are employed compared to unemployed patients in unadjusted analysis (Table 5). However, the amount of missing data should be considered when making inferences on the basis of these crude estimates. Pre-ART counselling was also significantly associated with decreased rates of LTFU in unadjusted analysis, while ART initiation counselling was not associated with LTFU.

**Table 5:** Unadjusted hazard ratios (uHR) and 95% confidence intervals (CI) from a shared frailty Cox proportional hazards model for the association between baseline covariates and the rate of loss to follow-up after starting antiretroviral therapy among newly diagnosed patients initiated on antiretroviral therapy within 14 days of enrolment (n = 2,482).

Characteristic		n/N	uHR	95% CI	P-value
**Local Government Area**					
	AMAC	287/932	1.00	-	-
	Buruku	127/367	0.92	0.42–1.99	0.83
	Calabar South	119/185	0.80	0.29–2.23	0.80
	Gwer West	100/330	0.78	0.28–2.21	0.64
	Ikeja	51/210	1.23	0.50–3.05	0.65
	Mushin	86/184	2.52	0.74–8.62	0.14
	Obio/Akpor	27/80	0.87	0.29–2.56	0.79
	Uyo	106/194	1.85	0.72–4.77	0.20
**Gender**					
	Female	605/1668	1.00	-	-
	Male	298/814	1.07	0.93–1.23	0.35
**Age**					
	16–24	182/394	1.00	-	-
	25–34	364/1006	0.77	0.64–0.92	0.004
	35–44	241/709	0.76	0.62–0.93	0.007
	45–54	84/276	0.66	0.51–0.85	0.002
	≥55	32/97	0.74	0.51–1.08	0.12
**Marital Status**^a^					
	Single	276/621	1.00	-	-
	Married	401/1246	0.75	0.64–0.88	0.0003
	Divorced	9/30	0.71	0.37–1.40	0.33
	Widowed	50/175	0.67	0.49–0.92	0.01
	Other	20/69	0.68	0.43–1.09	0.11
**Education**^b^					
	None	105/293	1.00	-	-
	Primary	147/364	0.94	0.70–1.25	0.65
	Secondary	265/660	0.90	0.68–1.19	0.47
	Post-Secondary	86/344	0.58	0.42–0.82	0.002
	University	2/17	0.27	0.07–1.13	0.07
**Employment Status**^c^					
	Unemployed	309/794	1.00	-	-
	Employed	392/1191	0.90	0.76–1.06	0.19
**Partner HIV Status**^d^					
	Negative	43/210	1.00	-	-
	Positive	30/181	0.78	0.48–1.25	0.30
**Pregnancy Status**^e^					
	No	557/1521	1.00	-	-
	Yes	41/128	0.86	0.62–1.19	0.37
**BMI**^f^					
	Normal range	381/1170	1.00	-	-
	Underweight	135/327	1.36	1.12–1.67	0.002
	Overweight	92/340	0.78	0.62–0.98	0.04
	Obese	40/141	0.83	0.59–1.15	0.26
**CD4 level**^g^					
	<200 cells/mm^3^	130/503	1.00	-	-
	201–350 cells/mm^3^	107/380	1.07	0.82–1.39	0.62
	351–500 cells/mm^3^	91/304	1.11	0.84–1.46	0.47
	> 500 cells/mm^3^	116/367	1.08	0.83–1.40	0.58
**WHO Stage**^h^					
	I	494/1593	1.00	-	-
	II	131/362	1.02	0.82–1.26	0.86
	III	218/419	1.52	1.24–1.87	<0.0001
	IV	14/35	1.39	0.81–2.39	0.24
**Functional Status**^i^					
	Working	795/2277	1.00	-	-
	Ambulatory	36/76	1.58	1.14–2.23	0.01
	Bedridden	6/9	2.89	1.28–6.54	0.01
**TB Status**^j^					
	No Sign of TB	636/1897	1.00	-	-
	Prior history of TB Tx	142/344	1.19	0.98–1.44	0.08
	On TB Tx	8/18	1.18	0.58–2.38	0.65
**Pre-ART Counselling**^k^					
	No	38/77	1.00	-	-
	Yes	741/2121	0.57	0.40–0.80	0.001
**ART Initiation Counselling**^l^					
	No	35/84	1.00	-	-
	Yes	748/2133	0.58	0.41–0.83	0.003

n = the total number of patients within the stratum who were lost to follow up out of the total N patients in that stratum

^a^ 341 patients did not have a relationship status documented

^b^804 patients did not have an educational status documented

^c^497 patients did not have an employment status documented

^d^2,091 patients did not have partner HIV status documented

^e^19 women did not have a documented pregnancy status

^f^BMI could not be calculated for 504 patients

^g^928 patients did not have a documented baseline CD4 cell count. Of note, 459/928 were lost to follow up (49%)

^h^73 patients did not have a WHO clinical disease stage documented

^i^120 patients did not have a functional status documented

^j^223 patients had no documentation of TB status

^k^284 patients had no documentation about receipt of pre-ART counselling

^l^265 patients had no documentation about receipt of ART initiation counselling

In predictive modelling for LTFU, stepwise regression analysis identified age group, CD4 cell count at baseline, and WHO stage at baseline as independent predictors of LTFU among *Test and Start* patients (Table 6). Specifically, those aged 55 years or older had an adjusted odds ratio (aOR) of 0.47 (95% CI, 0.23–0.93) of LTFU compared with those aged 16–24 years. Those with a CD4 count of >500 cells/mm^3^ had an aOR of 1.60 (95% CI, 1.14–2.25) of LTFU compared with those with a CD4 count <200 cells/mm^3^. Higher WHO disease stage was associated with stepwise increases in LTFU rates: Stage II with an aOR of 1.95 (95% CI, 1.41–2.69); Stage III with an aOR of 3.68 (95% CI, 2.70–5.01); and Stage IV with an aOR of 4.49 (95% CI, 1.93–10.43), compared with Stage I.

**Table 6:** Adjusted odds ratios (aOR) and 95% confidence intervals from generalized linear models using a binary outcome distribution for loss to follow up among test and start patients (n = 2,409).

Characteristic		aOR	95% CI	P-value
**Age**				
	16–24	1.00	-	-
	25–34	0.66	0.47–0.93	0.02
	35–44	0.62	0.43–0.89	0.01
	45–54	0.49	0.31–0.78	0.003
	≥55	0.47	0.23–0.93	0.03
**CD4 level**				
	<200 cells/mm3	1.00	-	-
	201–350 cells/mm3	1.40	0.99–1.96	0.05
	351–500 cells/mm3	1.47	1.03–2.10	0.04
	> 500 cells/mm3	1.60	1.14–2.25	0.007
**WHO Stage**				
	I	1.00	-	-
	II	1.95	1.41–2.69	<0.0001
	III	3.68	2.70–5.01	<0.0001
	IV	4.49	1.93–10.43	0.001

## Discussion

We found that approximately one in three HIV-positive patients in Nigeria who participated in the *Test and Treat* pilot were LTFU by 1 year after treatment initiation compared to approximately one in five HIV-positive patients who had been previously enrolled in care, though this finding is limited by the fact that those previously in care were perhaps engaged with the clinic for a longer time than *Test and Treat* patients and had survived to benefit from the change in guidelines removing CD4 cell count thresholds. However, implementing *Test and Treat* nationwide following the pilot model could significantly impair Nigeria’s ability to achieve the 90-90-90 targets and move toward epidemic control. Program data also show suboptimal retention in care and ART adherence. National expectations related to counseling and follow-up including frequent contact following treatment initiation and assessment of barriers to care could be considered at the facility level. Our findings provide valuable information to HIV service delivery implementing partners to help strengthen the health system and improve implementation of *Treat All* nationwide.

In this study, *Test and Treat* patients had lower baseline CD4+ cell counts and had more advanced disease at diagnosis, as evidenced by worse WHO disease stage, compared to patients who had been previously enrolled in care. This may indicate that people are still not presenting for testing until they are symptomatic, which could delay the population-level benefits of removing CD4+ cell count thresholds for treatment and the availability of rapid initiation interventions such as *Test and Treat*. Additionally, median ART initiation was on the day of diagnosis, and the *Test and Treat* patients had higher rates of LTFU than previously enrolled patients, which highlights the need for strategies to improve pre-ART counseling while balancing the drive for rapid initiation.

We found that the *Test and Treat* patients were less likely to consistently present at the point of service for scheduled visits than previously enrolled patients. Of the patients in this sample, only 45% had a visit in each of the four quarters of the first year of ART. Routine engagement in care is an important tool for epidemic control. Consistent visits would allow clinicians and care delivery staff to provide needed services such as continued adherence counselling, assessment of clinical symptoms, diagnosis of opportunistic infections or non-communicable disease comorbidities, and general preventive services. Inconsistent presentation at the point of service delivery could also contribute to disengagement from the care continuum. A recent study of patients in central or decentralized care in North Central Nigeria found differences in visit constancy and overall LTFU depending on where ART services were offered [13]. In this study, we found an association between visit constancy and LTFU. Most LTFU occurred in the first 30 days after initiating treatment. The proportion of LTFU among the *Test and Treat* patients was much larger than has been observed in previous evaluations of ART retention outcomes conducted before the adoption of the *Treat All* guidelines in Nigeria [14]. Identifying optimal intervals between patient visits and multiple measures of retention could help evaluate treatment programs [13, 15]. Our findings could be used to identify quality improvement initiatives to retain *Test and Treat* patients as the *Treat All* guidelines are implemented nationwide.

Patients LTFU were more likely to be younger, have a higher baseline CD4 cell count, a functional status of ambulatory or bedridden, and a WHO stage of III or IV compared to patients who were still alive at the time of review. High WHO stage and bedridden status may indicate that death is the reason for LTFU for a proportion of these patients. This finding indicates the importance of offering services for patients presenting with advanced disease and the need for continued active case-finding to diagnose and treat PLWH at early stages of disease. However, death is unlikely to explain all of the LTFU as observed by Charurat et al. [12]. The association of LTFU with high CD4 cell counts and ambulatory status may indicate that healthier patients are not prepared to adhere to ART. Additional support may be needed to educate patients about the need to remain in treatment to continue feeling well, and younger patients may require separate targeted support systems for retention. Implementing partners could consider working with the facilities they support to strengthen treatment preparation (particularly for immune competent asymptomatic patients), opportunistic infection diagnosis and advanced disease treatment, and community-based follow-up of patients who initiate treatment soon after diagnosis.

One opportunity for improving retention outcomes in Nigeria could be developing differentiated care models within the context of *Treat All*. Differentiated care refers to flexible service models developed to meet the needs of clients and clinics, particularly as more patients initiate treatment [9]. Service decentralization has been advocated by WHO for more than a decade [16], and has received increased attention recently as a way to expand access under the *Treat All* strategy. Community-based initiation and management of ART increases long-term retention rates in some settings [17–21]. To successfully implement *Test and Treat* approaches, flexible models of care are needed [13]. An evaluation of decentralized access to ART services in Swaziland demonstrated significantly reduced LTFU and general attrition among patients in decentralized settings compared to those at central facilities [22]. However, while overall retention was evaluated in these studies, it is unclear whether retained patients differed from non-retained patients in terms of routine visit adherence.

Viral load is considered the gold standard for monitoring adherence to treatment. However, viral load documentation was lacking within the selected facilities, suggesting that, for the vast majority of patients, clinicians do not have access to vital information related to treatment effectiveness or an objective measure of early treatment adherence. Our evaluation did not include care processes or treatment delivery models to enable us to understand the root cause of suboptimal utilization of viral load monitoring. A full mapping of the viral load process may be warranted to determine whether viral loads are being ordered, whether viral load orders are being processed, and whether the results are being returned to the facilities and then entered into the medical records. Nigerian health officials could consider implementing an alert system to notify clinicians when a patient is due for a viral load test and could consider interventions to increase patient-led demand for viral load testing. Additionally, data systems that allow the automatic upload of facility viral load results once entered into the laboratory system may mitigate this challenge going forward. Significant investment in laboratory infrastructure has been made in Nigeria, particularly since the beginning of PEPFAR [23–25]; however, linking, laboratory results, including viral load results, to patient records could improve clinical decision-making.

Given the high frequency of missing viral load results, we assessed adherence using pharmacy refill. After the first month of treatment, only approximately 30%–40% of patients refilled their prescription; the greatest increase in non-adherence to medication refill was in month 2 of treatment. Adherence to ART in the first several months is critical to control viral replication and prevent the development of drug-resistant mutations. Although actual adherence among these patients may be slightly higher due to earlier refill and thus maintenance of a buffer stock of pills, these findings suggest the need for programs to support medication adherence for patients earlier in disease progression or immediately after HIV diagnosis. *Test and Treat* as a long-term strategy for epidemic control must move beyond how quickly patients receive treatment as the primary measure of success. Early retention will be particularly important for Test and Treat to be successful. Patient and provider readiness, as well as the ability of the facility to absorb, manage, support, and follow a dramatically increased patient volume are also important factors to implement *Test and Treat* successfully [5, 6, 17, 18, 26].

Our study has several limitations. We were unable to assess whether patients classified as LTFU died after starting ART or migrated from the area. Additionally, *Test and Treat* has been implemented at other facilities, and it is possible that program maturity has improved retention outcomes. The amount of missing data for variables thought to be associated with LTFU precluded the use of methods such as multiple imputation to predict levels of the missing variables and thus limited their inclusion in adjusted models. Complete clinical data in patient medical records are needed for this analysis. Missing data represents a challenge not only to research and evaluation, but also the long-term longitudinal care of patients. Funders and Implementing Partners responsible for providing technical assistance at the facility level may wish to increase data quality improvement efforts to reduce the amount of missing clinical data. As programmatic reporting increasingly moves to electronic formats with direct health information exchange, data completeness checks for key indicators could be incorporated to help drive feedback to facilities to help improve data quality. Another limitation was that the study did not achieve the required sample size of 3,432 patients at a presumed 75% retention. An a priori assumption of the observed retention of 65% would have required an even larger sample size. Therefore, some important differences between comparison groups may not have met thresholds commonly used to define statistical significance. However, even without the desired power, this study still provides robust findings of significant predictors of retention and LTFU in Nigeria.

## Conclusions

In conclusion, although Nigeria, like many countries in sub-Saharan Africa, is eager to adopt and scale up *Treat All* and *Test and Treat*, our findings of persistent LTFU and lack of viral load results for medical decision-making underscore the importance of continuous quality improvement and monitoring patient outcomes. This is especially true given that more PLWH are now initiating ART. Continuous medical education about *Treat All* could help maintain the gains made toward epidemic control and avoid wasting resources. However, particular attention to early retention in the context of *Test and Treat*, which will be imporant Evaluating how *Test and Treat* strategies are implemented, monitored, and improved can help expand ART access to all successfully.

### PEPFAR/CDC author disclaimer

The findings and conclusions in this manuscript are those of the authors and do not necessarily represent the official position of the funding agencies. Use of trade names is for identification only and does not imply endorsement by the CDC or the U.S. Department of Health and Human Services.
